# ZTTK Syndrome Expanding the Cardiac Phenotype: A Case of Shone’s Complex With a Novel *SON* Variant and Focused Review of the Cardiac Manifestations in ZTTK Syndrome

**DOI:** 10.1155/crig/3473063

**Published:** 2026-09-16

**Authors:** John Coleman, Abdul L. Shakerdi, Veronika Dvorakova, Janna Kenny

**Affiliations:** ^1^ Department of Clinical Genetics, Children’s Health Ireland at Crumlin Children’s Hospital, Dublin, Ireland; ^2^ FutureNeuro, Department of Medicine, Royal College of Surgeons Ireland, Dublin, Ireland, duhs.edu.pk; ^3^ RDCat (Rare Disease Catalyst Consortium), University College Dublin, Dublin, Ireland, ucd.ie; ^4^ Trinity Translational Medicine Institute, St James’s Hospital & Trinity College Dublin, Dublin, Ireland; ^5^ Department of Dermatology, Children’s Health Ireland at Crumlin Children’s Hospital, Dublin, Ireland

## Abstract

Zhu–Tokita–Takenouchi–Kim (ZTTK) syndrome is a rare multisystem neurodevelopmental disorder caused by heterozygous variants in *SON*. Presented here is a case of a four‐year‐old female with multiple rare manifestations including a complex neurodevelopmental disorder, Shone complex and a simple capillary malformation. Genetic analysis confirmed a novel frameshift variant in *SON,* which was classified as pathogenic. The patient has epilepsy and moderate‐to‐severe global developmental delay. Her epilepsy is well controlled with levetiracetam monotherapy. A focused literature review was undertaken and found that cardiac defects are reported in an estimated 22% of cases to date. Obstructive left‐sided heart lesions are rarely reported, and this is the first reported case of Shone complex in the reported ZTTK patient cohorts. Loss‐of‐function variants in *SON* are implicated in abnormal RNA expression and splicing in genes involved in neural migration and metabolism. The literature review did not reveal an increased association between epilepsy in ZTTK patients and that in congenital heart disease compared with ZTTK patients without congenital heart disease, supporting the previous findings that *SON* is likely to be involved in multiple critical biological processes. Mitochondrial dysfunction was implicated in previous reports of loss‐of‐function *SON* variants and supported by the cardiac phenotype in previously reported cases.

## 1. Introduction

Zhu–Tokita–Takenouchi–Kim (ZTTK) syndrome is a rare multisystem neurodevelopmental disorder caused by heterozygous variants in the *SON* gene. ZTTK was first described in 2015, a neurodevelopmental disorder with variable manifestations [1–4]. Phenotypic features include seizures, congenital heart disease (CHD), visceral organ abnormalities, genitourinary abnormalities, short stature, musculoskeletal abnormalities, developmental delay, structural brain abnormalities and variable dysmorphic features [5]. Disease‐causing variants in *SON* can cause disruption of transcription/RNA splicing. Haploinsufficiency results in dysregulated neural migration and dendritic abnormalities [6]. Frameshift, nonsense and missense variants have been reported [5, 6]. It is ubiquitously expressed in different tissues and organs [7]. Recently, patients with ZTTK have been described with myocardial injury, cardiomyopathy and metabolic stroke, suggesting that abnormalities of mitochondrial function may contribute to the pathophysiology [8, 9].

Shone complex is a rare CHD comprising a constellation of left‐sided obstructive cardiac lesions including supravalvular mitral membrane, parachute mitral valve, subaortic stenosis and coarctation of the aorta [10]. It is a rare form of CHD, representing approximately 0.7% of defects [11]. It is commonly diagnosed in infancy but can be diagnosed at various stages of life from the prenatal period into adulthood. A single mechanism or genetic aetiology has not been confirmed to cause this condition [12]. The association of Shone complex with ZTTK syndrome has not been previously reported.

We present a case of a female paediatric patient with multiple rare disease manifestations including ZTTK syndrome, Shone complex and a simple capillary malformation. This includes a focused review of the cardiac phenotype associated with ZTTK syndrome to examine the evidence and identify associated or causal factors.

## 2. Patient Information

A 4‐year‐old girl was referred to the clinical genetics team because of a complex congenital heart condition, Shone complex. The patient had multiple comorbid medical needs including epilepsy, global developmental delay, a simple capillary malformation affecting the face and a duplex left kidney. She was born at term following a spontaneous conception. Prenatal history included maternal gestational diabetes mellitus that was well controlled with diet. There was no maternal medication use or teratogen exposure. She weighed 3.03 kg (18th centile) at the time of birth. Thirty minutes following birth, the patient became lethargic and cyanotic in appearance and was admitted to the neonatal intensive care unit, where she remained CPAP‐dependent for the first week of life. She was subsequently diagnosed with Shone complex on echocardiogram during this admission. The patient has never required intervention for this and remains well under regular cardiac surveillance. Her parents are nonconsanguineous. She has two healthy older brothers. There is no close family history of cardiac, neurological or neurodevelopmental disorders.

Her seizures began at the age of 1 year, when she presented with status epilepticus secondary to a generalised tonic–clonic seizure. She had abnormalities on EEG and was commenced on levetiracetam. Since then, she has had two further seizures related to suspected infection. There was no evidence of clinical immunodeficiency or recurrent severe infections. The patient has a facial capillary venous malformation present since birth. She had delayed tooth eruption, and her family noted poor nail growth.

The patient has global developmental delay across all domains. Her speech is delayed. At the age of 4 years, she could use 2–3 words. She is unable to walk but could crawl. She could feed herself with her fingers but could not grasp a pencil. She was not toileting independently. She had a happy demeanour and is stranger aware. She could not wave or greet. Her hearing and vision were normal.

At the age of 6 years, the family was reviewed again. The patient was stable medically, and her epilepsy was well controlled on levetiracetam monotherapy. Her current seizure type was absence seizures. Her cardiac function remained stable. She continued to make developmental progress and could walk with a walking aid. She was not speaking sentences but could say 2 or 3 words with meaning (naming family members). Her family described her as ‘sweet natured’. She was sleeping throughout the night. She was not yet toileting independently.

## 3. Clinical Findings

**FIGURE 1:**
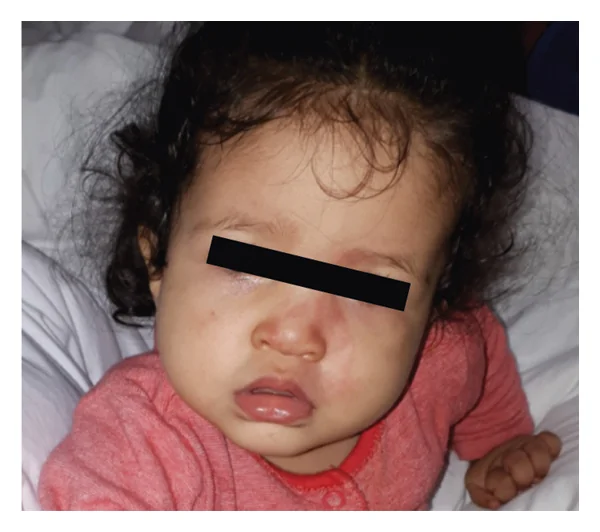
Photograph taken at 1 year of age. A capillary venous malformation, down‐slanting palpebral fissures, long eyelashes, a flat and widened nasal bridge, sparse eyebrows and a low anterior hairline were noted.

**FIGURE 2:**
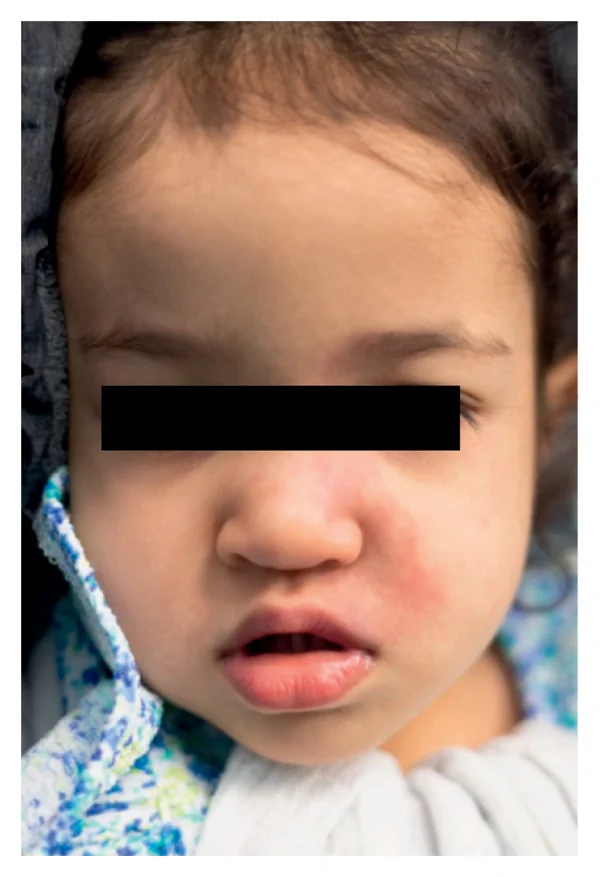
Photograph taken at 4 years of age, front view. A capillary venous malformation less prominent than at age 1, a broad nasal bridge, a broad and flattened nasal tip, hypertelorism, prognathia, long eyelashes, down‐slanting palpebral fissures, ptosis, a prominent lower lip and a thin lateral eyebrow were noted.

**FIGURE 3:**
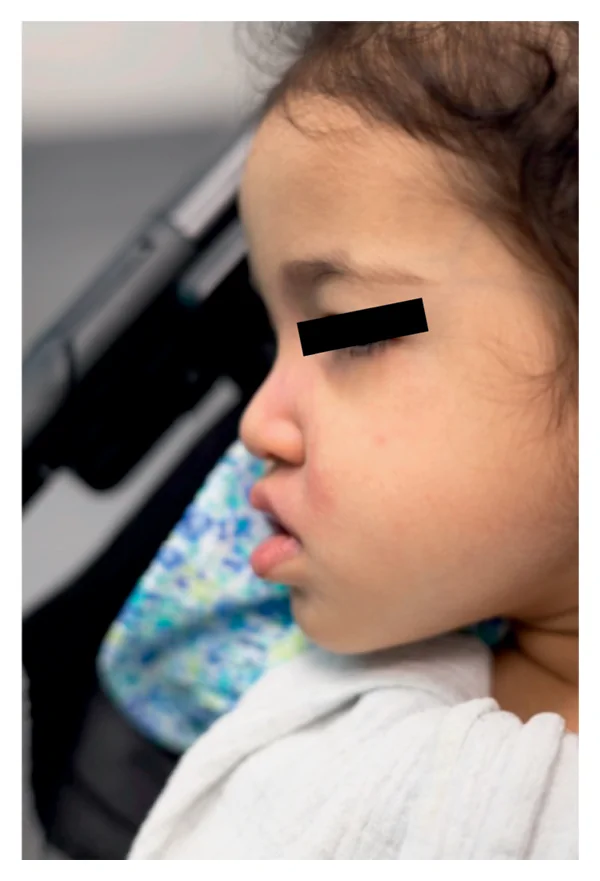
Patient side profile showing prognathia and a venous malformation.

On examination, the patient had down‐slanting palpebral fissures. She had mild hypertelorism with a flat nasal bridge. She was noted to have microdontia, micrognathia and long eyelashes. There was a low anterior hairline, and sparse lateral eyebrows were noted. The ears were posteriorly rotated. There was a capillary venous malformation across the middle section of the face including the nose and over the distribution of the face on the left side of the face involving the maxillary region but sparing the forehead (see patient Figures 1, 2 and 3). There was a single café au lait patch 2 × 4 cm on the left forearm. There was notable joint hypermobility. Ptosis was noted as she grew older but not on initial examination.

## 4. Diagnostic Assessment

Renal ultrasound, 2019: The left kidney had a duplex configuration; otherwise, the kidneys had no other abnormal findings.

Brain MRI, 2020: The brain parenchyma was normal with no intracranial abnormality detected.

EEG, 2020: The EEG showed persistent rhythmic, high‐amplitude slowing with occasional broad complex activities over the left posterior greater than mid temporal region. This confirmed a focal cortical disturbance in the left posterior greater than mid temporal region.

Array CGH, 2020 (GRCh37): Arr(1‐22,X)x2. No diagnostic abnormality was detected across 60,000 probes using the Agilent SurePrint G3 platform.

Echocardiogram, 2023: The echocardiogram showed Shone complex. The atrial and ventricular were septa intact. There was trace mitral and tricuspid regurgitation. The descending aorta was mildly hypoplastic.

Ophthalmology assessment showed exotropia of approx. 30 dioptres for near and on cover testing, with exotropia measuring 40 dioptres for distance. No obvious field defects were detected, nor was there a refractive error.

A trio exome analysis was completed using Illumina sequencing with an external laboratory partner. Sequencing included analysis of the coding regions and conserved splice sites of 23,244 genes by next‐generation sequencing (Twist Core Human Exome/Illumina NextSeq/NovaSeq). Parentage was confirmed. CNV calling was completed with the assay but does not detect balanced arrangements. Small variant detection (SNV and indels) sensitivity in the testing was > 99% at a read depth of more than 20x, although mosaic variant calling is limited. Variants are classified using the ACMG classification [13]. A de novo novel pathogenic variant in *SON* was identified, confirming a diagnosis of ZTTK syndrome (see Table 1).

**TABLE 1:** Results of genetic testing.

Gene	Zygosity	Inheritance	HGVS description	Genomic coordinates	Classification
*SON*	Heterozygous	De novo	NM_138927.4:c.4723_4729del p.(Arg1575Tyrfs∗46) (NP_620305.3)	Chr21(GRCh38):g.33553954 _33553960del	Pathogenic

## 5. Literature Review

A focused literature review was completed using PubMed Central. Search terms included ‘ZTTK’ and ‘ZTTK syndrome’. This yielded 51 cases of interest (see Table 2). Duplicate reports from review papers or those without a human patient cohort were excluded. To describe the cardiac phenotypes in ZTTK, a focused strategy was undertaken to collect phenotype and reported clinical data for those with cardiac manifestations of the disorder during completion of the literature review (see Table 1). The variant detected in the patient described above has not previously been reported in the literature.

**TABLE 2:** Focused review of the cardiac phenotype reported with ZTTK. + indicates that the feature was present, and − indicates that the feature was not reported/absent.

	Cardiac phenotype	Cardiac Tx	CNS features (epilepsy/developmental disorder)	Genitourinary abnormality	Dysmorphic features	Skeletal abnormalities	Eye abnormality	Other
Our proband/case	Shone’s complex	Surveillance only	+	−	+	−	+	Dental and nail abnormality, facial haemangioma
PMID								
32705777 [14]	Hypoplastic left heart	Not described	+	+	+	+	+	Duodenal atresia, intestinal malrotation
32045494 [15]	ASD + VSD	Surgical intervention	+	+	+	+	+	Dental and nail abnormality
34331327 [16]	ASD	Not described	+	+	+	−	+	−
34331327 [16]	ASD	Not described	+	+	+	+	+	Hearing loss, craniosynostosis
34521999 [5]	VSD	Not described	−	+	+	+	+	−
34521999 [5]	ASD + VSD	Not described	+	+	+	+	+	Dental, nail and hair abnormality
35740806 [17]	Tetralogy of Fallot, right heart dilatation, pulmonary valve regurgitation Grade III, mild stenosis of the left branch of the pulmonary artery	Surgical intervention	+	+	+	+	+	−
36175752 [18]	Ductus arteriosus and ASD	Not described	+	+	+	+	−	Pulmonary abnormalities
36540882 [19]	ASD, right cavity mildly dilated	Not described	+	+	+	+	−	Dental anomalies/precious dentition, retractile testicles, flat angioma forehead
37168776 [10]	Cardiac abnormalities, not specified	Not described	+	+	+	+	+	Gastrointestinal abnormalities
38566089 [9]	Three episodes of myocardial injury, myocarditis	Metoprolol, intravenous coenzyme Q10.	+	+	+	−	+	−

All cases described had reported developmental delay, and 49% had epilepsy (25/51). Cardiac abnormalities were described in 22% of cases (11/51). Structural heart defects included tetralogy of Fallot (1/11), atrial septal defect (ASD) (4/11), ventricular septal defect (VSD) (1/11), ASD + VSD (2/11), hypoplastic left heart syndrome (1/11), recurrent myocardial injuries (1/11) and unspecified cardiac abnormalities (1/11). The genotype described for 10/11 of these cases was predicted as loss of function, and 1/11 was unspecified. Epilepsy was present in 45% of cases (5/11).

## 6. Discussion

This case is a novel report of ZTTK associated with Shone complex. Shone complex is associated with left‐sided obstructive heart lesions [10]. For those cases reported in the literature, the most prominent lesions are septal defects (64%), hypoplastic left heart syndrome [14] and tetralogy of Fallot [17], and these were the two complex lesions reported to date. While some features of obstructive heart lesions [14] and valvular issues have been reported [17], Shone complex or other features such as subaortic stenosis, coarctation, parachute mitral valve or supravalvular mitral ring were not revealed in our review of the literature. Epilepsy was not overrepresented in those with CHD in the cohort (45%) compared with epilepsy in the entire cohort (49%). Previous reports describe abnormal expression of RNA in genes critical for neural migration in individuals with loss‐of‐function *SON* variants [4]. Multiple additional congenital abnormalities were reported across all cases in the literature, which supports the theory that *SON* may be important in various embryonal and developmental processes.

One other case from the literature with CHD [19] has an angioma present on the forehead. Although it is difficult to compare an angioma with the capillary malformation in our patient, this, in combination with the various congenital and cardiac anomalies represented in ZTTK, may be suggestive that the responsible gene may affect the vascular regulation processes during embryonal development. Most commonly, simple capillary malformations are associated with somatic variants in *GNAQ, GNA11, GNA14* and, more rarely, germline or somatic syndromic (e.g., *PIK3CA*) and nonsyndromic conditions [20]. Given the location of the capillary lesion, limited management implications and complexity of our case, a somatic biopsy was not pursued. It is plausible that this phenomenon may be due to a process unrelated to ZTTK.

The variant in our case is a frameshift variant NM_138927.4:c.4723_4729del, and it has not been reported in the literature. Loss of function is a known mechanism for ZTTK [21]. One of the most approximate reported variants to ours in the literature involves a case with a frameshift SON:c.3852_3856delGGTAT variant, detected in a 7‐year‐old with recurrent myocardial injury, intellectual disability and syndromic features. On three occasions, they presented with elevated myocardial enzyme spectrum indicators and T‐wave changes or ST‐T changes on ECG. Echocardiogram on each occasion did not detect other structural or valvular lesions, and interval improvements in the ECG were seen [9]. The next most approximate and comparable variant in the literature is a de novo frameshift variant SON: c.5334_5335delAG in a female. She had neurological signs including mild delayed myelination, enlarged bilateral ventricles and widened frontotemporal extracerebral space. She had esotropia and mild facial abnormalities with a flat nasal bridge and a short nose. She showed no abnormalities in the heart, genitourinary or skeletal systems. There was no evidence of seizures or abnormality noted on EEG in this patient either [22]. This variant is located further toward the 5′ end of the DNA compared with the variant detected in our patient and may contribute to the phenotypic difference. Trio whole‐exome sequencing in the case did not detect additional variants to explain the phenotype. The analysis pipeline filters to include variants that are de novo, predicted high impact variants, compound bilallelic variants, splicing variants, and panels are applied for genes associated with cardiovascular presentations.

It is hypothesised that ZTTK syndrome results in abnormal splicing of mRNAs of cardiac transcription factors or structural proteins [23]. Abnormal expression and alternative splicing in 11 genes were recorded in blood samples obtained from individuals with loss‐of‐function *SON* variants. These were genes critical for neural migration and metabolism [4]. *IDH2* is one example of a downregulated gene. It has previously been associated with cardiac hypertrophy and enhanced oxidative stress in the mitochondria [24]. It is possibly contributory to recurrent myocardial injuries described in ZTTK [9]. *PCK2* is another gene reported to have abnormal expression [4]. *PCK2* has previously been implicated in cardiomyocyte metabolism, and abnormal RNA splicing of this gene may be implicated in the development of cardiac hypertrophy or may be vital in other developmental cardiac processes [24]. In our case, it is possible that abnormal early development of cardiomyocytes caused downstream structural events, which led to the development of Shone complex [4, 24, 25]. Other examples of RNA splicing regulatory genes causing cardiac phenotypes (congenital heart defects and cardiomyopathy) include *EFTUD2* [26], *RBM20* [27, 28], *TXNL4A* [29] and *SF3B4* [30].

From a management perspective, ZTTK syndrome requires a multidisciplinary approach. Cardiac care is one aspect, alongside neurologic, developmental, ophthalmologic, orthopaedic and gastroenterological care. Regarding cardiac management, no ZTTK‐specific guidelines have been described in case reports. In reports where therapeutic intervention was described [9, 15, 17, 19], lesions have mostly been addressed according to standard paediatric cardiology practices. Hudec and Kosinova [17] report that careful preoperative assessment, including cardiac assessment, is crucial in these patients. Individualised care planning may be required, particularly for hypotonia and dysmorphism, which can affect airway and operative care. In clinical practice, considerations for investigations, including cardiac imaging, and assessment for features of mitochondrial dysfunction may be worthwhile in symptomatic cases. This may be an important consideration given the implication of this gene in mitochondrial metabolic regulation; however, the evidence regarding *SON* variants continues to emerge [4, 9, 24, 25].

**Nomenclature**
Array CGH: Array‐based comparative genomic hybridisation
ASD: Atrial septal defect
CHD: Congenital heart defect
Chr: Chromosome
CNS: Central nervous system
CPAP: Continuous positive airway pressure
DNA: Deoxyribonucleic acid
EEG: Electroencephalogram
MRI: Magnetic resonance imaging
PMID: PubMed identification number
RNA: Ribonucleic acid
VSD: Ventricular septal defect
ZTTK: Zhu–Tokita–Takenouchi–Kim

## Author Contributions

John Coleman: paper conceptualisation and drafting, obtained informed patient consent and review of the relevant literature. Abdul L. Shakerdi: review of the relevant literature and manuscript drafting. Veronika Dvorakova: patient care provider and manuscript editing. Janna Kenny: manuscript drafting and editing, patient care provider and project supervision.

## Funding

Formal salary support for research‐related activities for the corresponding author is provided through the Rare Disease Research Catalyst Consortium (RD Cat), which is funded by the Health Research Board (HRB), Ireland.

## Consent

This report was completed with informed patient consent including consent for publication of clinical photography.

## Conflicts of Interest

The authors declare no conflicts of interest.

## Data Availability

Additional clinical data are available on request from the corresponding authors.
